# 

**Published:** 1888-09

**Authors:** 


					﻿nwunejinmkmopm,pe,rp,mpm.,p[m,.p5[.,;p[
Vol. IV.
SEPTEMBER. 1868
No. 3
DANIEL'S
MEDICAL
JOURNAL
sklnklml,lpm,l;r,,,okom,l, l;d';,t.'[pleojkoomomoioytfat6fyggoophk,op
sknomkinmkemromop,lm ,rl;rt;l.lp
				

